# Pusa Basmati 1121 – a rice variety with exceptional kernel elongation and volume expansion after cooking

**DOI:** 10.1186/s12284-018-0213-6

**Published:** 2018-04-09

**Authors:** Vijaipal Singh, Ashok Kumar Singh, Trilochan Mohapatra, Gopala Krishnan S, Ranjith Kumar Ellur

**Affiliations:** 1 E-9, Sector 40, Noida, UP 201303 India; 2 Division of Genetics, ICAR-Indian Agricultural Research Institute, New Delhi, 110012 India; 3 Indian Council of Agricultural Research (ICAR), New Delhi, 110001 India

**Keywords:** Basmati rice, Aroma, Grain and cooking quality, Linear cooked kernel elongation, Pusa Basmati 1121, Molecular mapping, Genomics

## Abstract

Pusa Basmati 1121 (PB 1121) is a landmark Basmati rice variety having Basmati quality traits introgressed from traditional Basmati varieties such as Basmati 370 and Type 3. It was released for commercial cultivation in 2003. It possesses extra-long slender milled grains (9.00 mm), pleasant aroma, and an exceptionally high cooked kernel elongation ratio of 2.5 with a cooked kernel length of up to 22 mm, volume expansion more than four times, appealing taste, good mouth feel and easy digestibility. Owing to its exceptional quality characteristics, it has set new standards in the Basmati rice market. The cumulative foreign exchange earnings through export of PB 1121 since 2008 have been US$ 20.8 billion, which has brought prosperity to millions of Basmati farmers. During 2017, the farmers cultivating PB 1121 earned on an average US$ 1400/ha as against US$ 650/ha cultivating traditional Basmati, making it a highly profitable enterprise. Currently, PB 1121 is grown in ~ 70% of the total area under Basmati rice cultivation in India. It is the most common Basmati rice variety in rice grain quality research for developing mapping populations, genetic analyses and molecular mapping of Basmati quality traits. Additionally, it has been widely used in the Basmati rice breeding program across India, because of its superior quality attributes. This article presents an account of development of PB 1121, its major characteristic features and its flagship role in heralding a Basmati rice revolution. The prospective role of PB 1121 in Basmati rice improvement and future Basmati rice research as a whole is also presented.

**Electronic supplementary material:**

The online version of this article (10.1186/s12284-018-0213-6) contains supplementary material, which is available to authorized users.

## Background

Basmati is one of the unique specialty rice varieties, which has been cultivated for centuries at the foot of Himalayan mountain ranges. Basmati rice was a predominant constituent of the rich and royal menus. Basmati rice has a harmonious combination of defined kernel dimensions, appealing aroma, fluffy texture of cooked rice, high volume expansion during cooking, linear kernel elongation with minimum breadth-wise swelling, palatability, easy digestibility and longer shelf-life (Singh et al. 1988). In India, Basmati rice is primarily grown in the Indo-Gangetic region of north-western region comprising the seven states Punjab, Haryana, Himachal Pradesh, Uttarakhand, Jammu and Kathua districts of Jammu and Kashmir, and 27 districts of western Uttar Pradesh. This region has been earmarked as the Geographical Indication (GI) for Basmati rice and the GI status has been conferred to Basmati rice in 2016 (GI No. 145 of the Geographical Indication Registry, Government of India, *vide* certificate No. 238 dated 15.02.2016). India is the largest cultivator and exporter of Basmati rice, followed by Pakistan. Basmati rice from the Indian subcontinent is highly prized in the international market for its unique grain, cooking and eating quality.

The name ‘Basmati’ is likely to have originated from the Sanskrit word, *bas* from ‘*vasay*’ connoting aroma; and *mati* from *mayup* meaning ingrained from the beginning. Common usage could have resulted in change of *vas* to *bas* resulting in Basmati (Singh and Singh 2009). The earliest mention of the word Basmati has been made in the epic “Heer and Ranjha” composed by the Punjabi poet Varish Shah in 1766. In a compilation of “Races of Rice in India”, Basmati has been differently spelled (Bansmatti, Bansmutty, Bansmati, Bansmuttee and Basmatee) and described as “A race of rice cultivated throughout the erstwhile Punjab” (comprising Punjab, Haryana and Himachal Pradesh), Delhi, Uttrakhand, parts of Uttar Pradesh and Jammu and Kashmir (Anonymous, 1910).

**Fig. 1:**
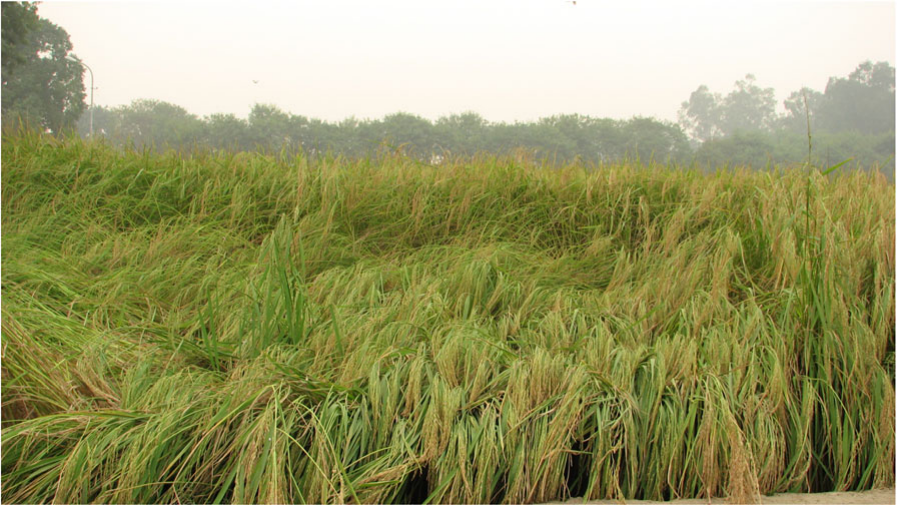
Lodging in the traditional Basmati rice variety, Type 3 at maturity due to tallness

Traditional Basmati varieties are tall, prone to lodging, photoperiod and temperature sensitive and very low yielding (Fig. 1). Therefore, their production and productivity were limited (Singh et al. 2011). Initial research efforts on Basmati rice improvement based on germplasm collection, evaluation and selection led to the identification of a line number 370/27, released as “Basmati 370” for cultivation in 1933 which was the most popular variety for the purpose of export until the late 1980’s (Ramaiah and Rao, 1953). However, Basmati rice did not acquire a status of a commercial enterprise until the ICAR-Indian Agricultural Research Institute (ICAR-IARI), New Delhi popularly known as “Pusa Institute”, took the lead to improve the plant type through systematic research spanning five decades starting from the late 1960s (Siddiq et al. 2012). Pioneering research work was carried out at the ICAR-IARI, New Delhi during the 1970s on standardization of protocols for estimating various Basmati quality parameters and analyzing their inheritance pattern (Singh et al., 2004).

The first breakthrough in developing higher-yielding Basmati rice came in 1989, with the release of Pusa Basmati 1 (IET 10364). It was the world’s first semi-dwarf, photoperiod-insensitive and high yielding Basmati rice variety developed from the cross between Pusa 150 and Karnal Local. Pusa 150 is a breeding line which was derived through a convergent breeding approach involving several high yielding non-aromatic rice varieties such as Taichung Native 1, IR8, IR22 etc., and traditional Basmati rice variety, Basmati 370, which was used as donor for quality traits. Karnal Local was a selection from the traditional Basmati rice collection, Haryana Basmati Collection 19 (HBC 19) from the Karnal district of Haryana with better grain and cooking quality, which was later released as Taraori Basmati in 1996 (Singh et al. 2004). It was one of the most widely used parents by rice breeders for grain, cooking and eating characteristics. The International Rice Research Institute (IRRI) used it in as many as 249 crosses (Singh and Singh, 2010). Extra-long slender aromatic grains, less cooking time and higher linear cooked kernel elongation of freshly harvested rice coupled with a potential grain yield of 5.0 tons/ha and medium early duration (135–140 days seed to seed maturity) made Pusa Basmati 1 the most sought after variety by the farmers, exporters and consumers. During the 1990’s, ICAR-IARI gave priority to longer grain length and higher linear cooked kernel elongation during cooking in addition to other Basmati attributes and a decade of focused work resulted in the development of Pusa Basmati 1121.

## Breeding history

**Fig. 2:**
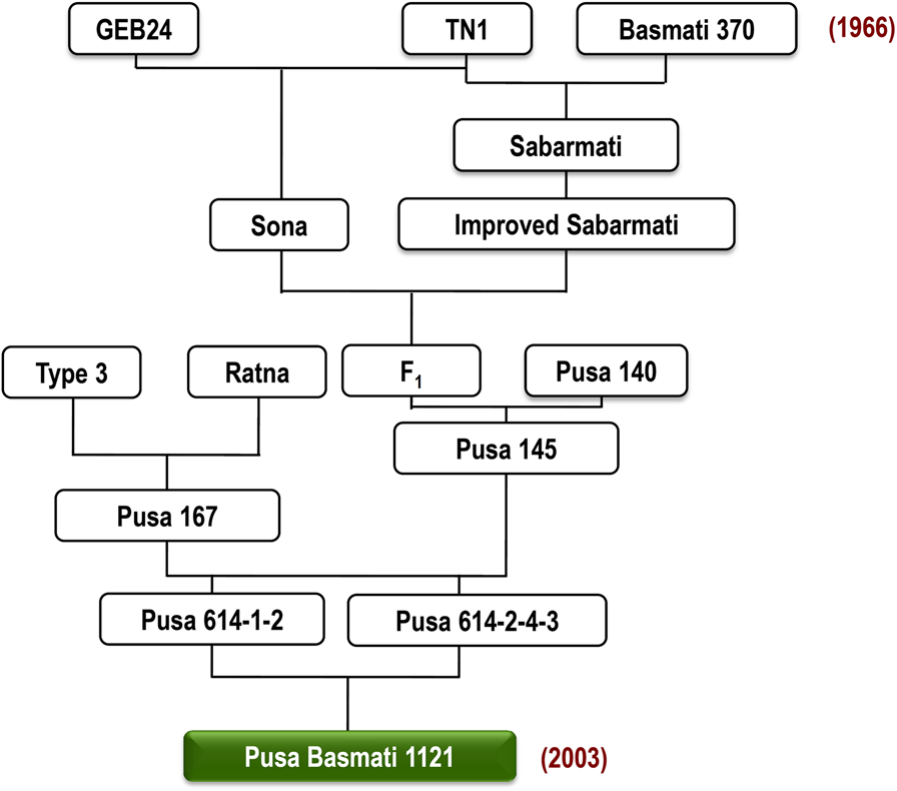
Lineage of PB 1121 showing the contribution of several varieties including the traditional Basmati rice varieties Basmati 370 and Type 3. Values in parentheses are the year in which crossing was initiated (1966) and the year of release of the variety (2003)

A brief account of the development of Pusa Basmati 1121 has been documented by Singh et al. (2002). During *Kharif* (rainy season/ wet season) 1991, a cross involving advanced breeding lines, Pusa 614–1-2 and Pusa 614–2–4-3, derived from traditional Basmati varieties such as Basmati 370 and Type 3 in it pedigree, was made with the objective of improving grain length and linear cooked kernel elongation at ICAR-IARI, New Delhi (Fig. 2). In *Kharif* 1992, a large number of single plant selections was made from the F_2_ population. One of these possessed longer milled rice kernels and showed exceptionally high linear cooked kernel elongation. Although the linear cooked kernel elongation and aroma were stable, there was still variation for brown rice grain dimension, breadth and texture of cooked rice and bursting during cooking in the subsequent generations. Several rounds of stringent phenotypic selection in the following generations helped in stabilizing the line for milled rice kernel length, aroma and its exceptional cooking quality. One of the superior lines, Pusa 1121–92–8-13-3 (IET 18004) was entered into the Basmati trials (presently the National Basmati Trials) under the All India Coordinated Research Project on Rice in 2002. It was released for commercial cultivation in the national capital region of Delhi in 2003, and subsequently for the states of Punjab and Haryana in 2008 (*vide* gazette notification no. S.Q. 2547(E), Ministry of Agriculture, Government of India dated 29th October, 2008).

The superior linear cooked kernel elongation in PB 1121 was derived from parents Basmati 370 and Type 3, which were used as donors for grain and cooking quality traits. The accumulation of favorable loci for extra-long grain and exceptionally high linear cooked kernel elongation was possible through transgressive segregation resulting from selective inter-mating of the sister lines showing better linear kernel elongation in the segregating generations. The lineage of PB 1121 **(**Fig. 2**)** clearly shows that as many as 13 rice varieties/enhanced germplasm, including the traditional Basmati rice varieties such as Basmati 370 and Type 3 were used to bring together the favorable alleles at multiple loci for agronomic, grain and cooking quality characteristics in development of PB 1121.

## Key agronomic and quality traits

**Fig. 3:**
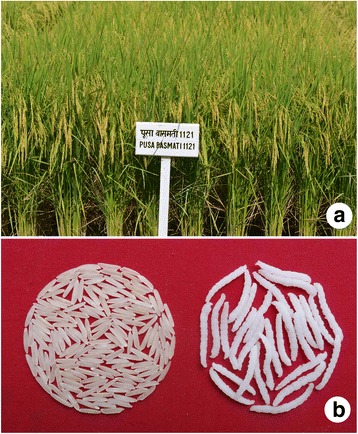
**a** Field view of the plot of PB 1121 at ICAR-IARI, New Delhi, (**b**) Milled rice and cooked rice kernels of PB 1121

PB 1121 is a semi-dwarf rice variety with a plant height ranging from 110 to 120 cm (Fig. 3a). The *sd1* allele for reduced plant height was derived from Taichung Native 1. It has a seed to seed maturity of 140–145 days in the Basmati growing regions of India. PB 1121 has a yield potential of up to 5.5 tons per hectare. It can produce 18–20 tillers per plant at a spacing of 20 cm × 15 cm and around 350–400 panicles per m^2^. It possesses well exserted long panicles, ranging from 26 to 28 cm. The number of filled grains per panicle ranges from 105 to 110 and the 1000-grain weight of the fully mature grains is 27.0–28.0 g (at 14% moisture). Since Basmati grains are extra-long and slender in appearance, they are prone to breakage during machine harvesting. Therefore, manual threshing in Basmati rice is preferred as it results in reduced broken grains, high head rice recovery and better price realization for farmers as compared to a machine harvested crop. PB 1121 has easy threshability, which is advantageous for manual threshing by the farmers.

The key grain characteristics of PB 1121 are presented in Table 1. It has extra-long slender grains with a milled rice kernel length averaging 9.00 mm, a breadth of 1.90 mm and an L/B ratio of 4.74 (Fig. 3b). Despite extra- long slender grains, it has an appreciable head rice recovery of 54.5% with a hulling and milling percentage of 75.0 and 70.5, respectively. The brown rice of PB 1121 is tan in colour. The alkali spreading value (ASV) score is 7 and the amylose content is 22.0%. The linear cooked kernel length of PB 1121 is another unique feature, which is the highest ever recorded in rice germplasm, ranging from 21.0 to 21.5 mm with minimum breadthwise swelling (only 2.45 mm). The kernel elongation ratio of 2.70 is also the highest ever known for any rice line. It also possesses strong aroma and has been rated as excellent cooking in the panel test (Additional file 1: Table S2).

**Table 1:** Grain and cooking quality characteristics of improved Basmati rice variety PB 1121 compared to Basmati 370, Taraori Basmati and Pusa Basmati 1

Variety	Milling%	HRR%	KLBC (mm)	KBBC (mm)	KLAC (mm)	ER
Basmati 370	72.5	53.0	6.89	1.85	13.40	1.94
Taraori Basmati	69.0	49.9	7.15	1.78	13.97	1.95
Pusa Basmati 1	67.0	48.5	7.38	1.80	14.75	2.00
PB 1121	70.5	54.5	8.00	1.90	21.50	2.69

HRR-Head rice recovery, KLBC-Kernel length before cooking, KBBC - Kernel breadth before cooking, KLAC-Kernel length after cooking, ER-Elongation ratio (Source: Singh et al. 2002)

## Improvement in PB 1121 as compared to traditional Basmati rice varieties

### Duration and productivity

**Fig. 4:**
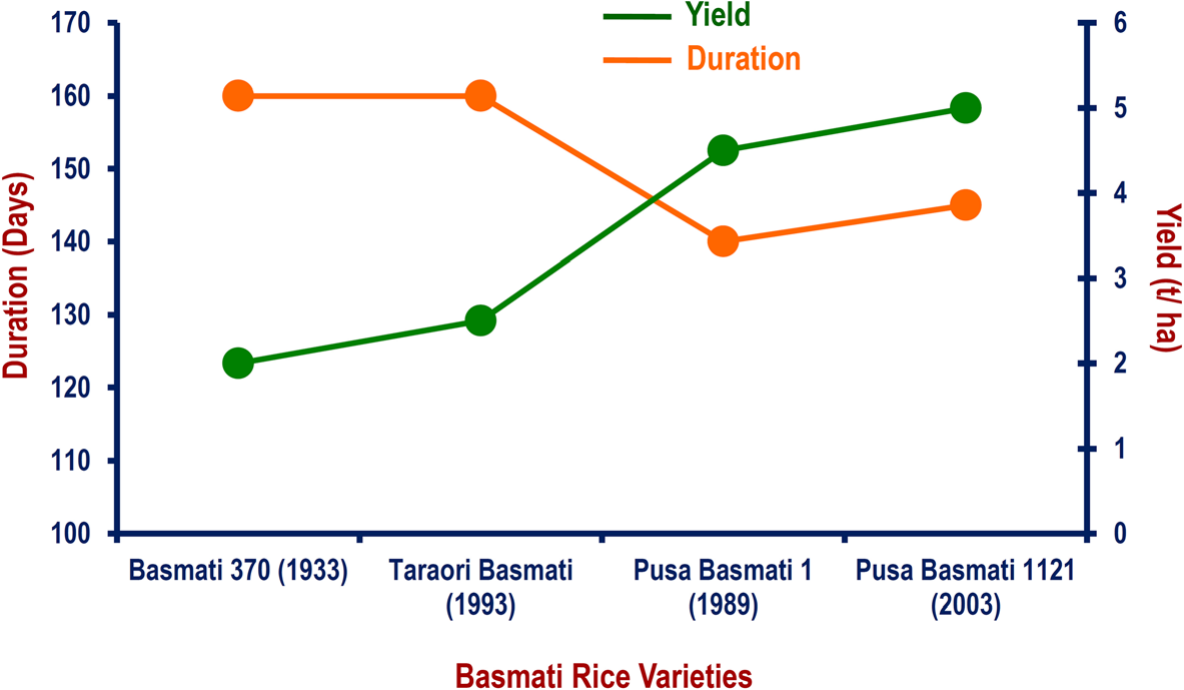
Duration and grain yield of PB 1121 as compared to Basmati 370, Taraori Basmati and Pusa Basmati 1; Year of release of the variety is indicated in the parentheses

Basmati rice research work carried out at ICAR-IARI, New Delhi has resulted in reduction of growth duration from 160 days in traditional Basmati to 140–145 days. Combined with the doubling of the yield from 2.5 t/ha to 5.0 t/ha, this results in higher per day productivity and saving of inputs like fertilizer, irrigation and pesticides thus bringing down the cost of cultivation (Fig. 4) and giving more turnaround time to farmers for timely sowing of wheat, which is a very important winter season crop in the rice-wheat cropping system of north-western India.

### Grain and cooking quality

**Fig. 5:**
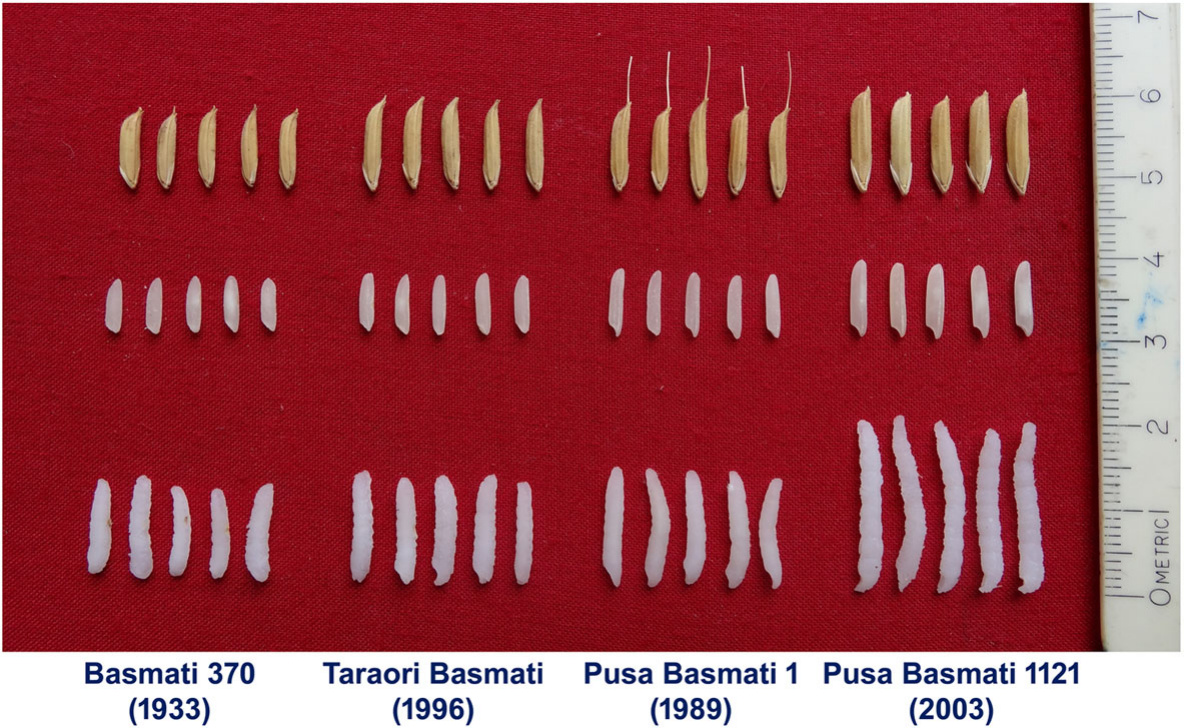
Gradual improvement in grain and cooking quality of Basmati rice varieties: a journey of decades

Systematic breeding efforts at ICAR-IARI, New Delhi has helped in improving the grain and cooking quality in PB 1121 as compared to other Basmati rice varieties released earlier (Table 1, Fig. 5). The most striking characteristic feature of PB 1121 as compared to other Basmati rice varieties is its kernel length after cooking, which is 21.50 mm. There has been an improvement in the milled rice kernel length to 8.00 mm as compared to 6.89 mm in Basmati 370. As a result of its improved kernel elongation, the volume expansion in the cooked rice is more than 4.0 times. This helps in realizing more cooked rice volume with lesser milled rice, a trait most preferred in the household as well as the hotel industry.

## Adoption and impact

**Fig. 6:**
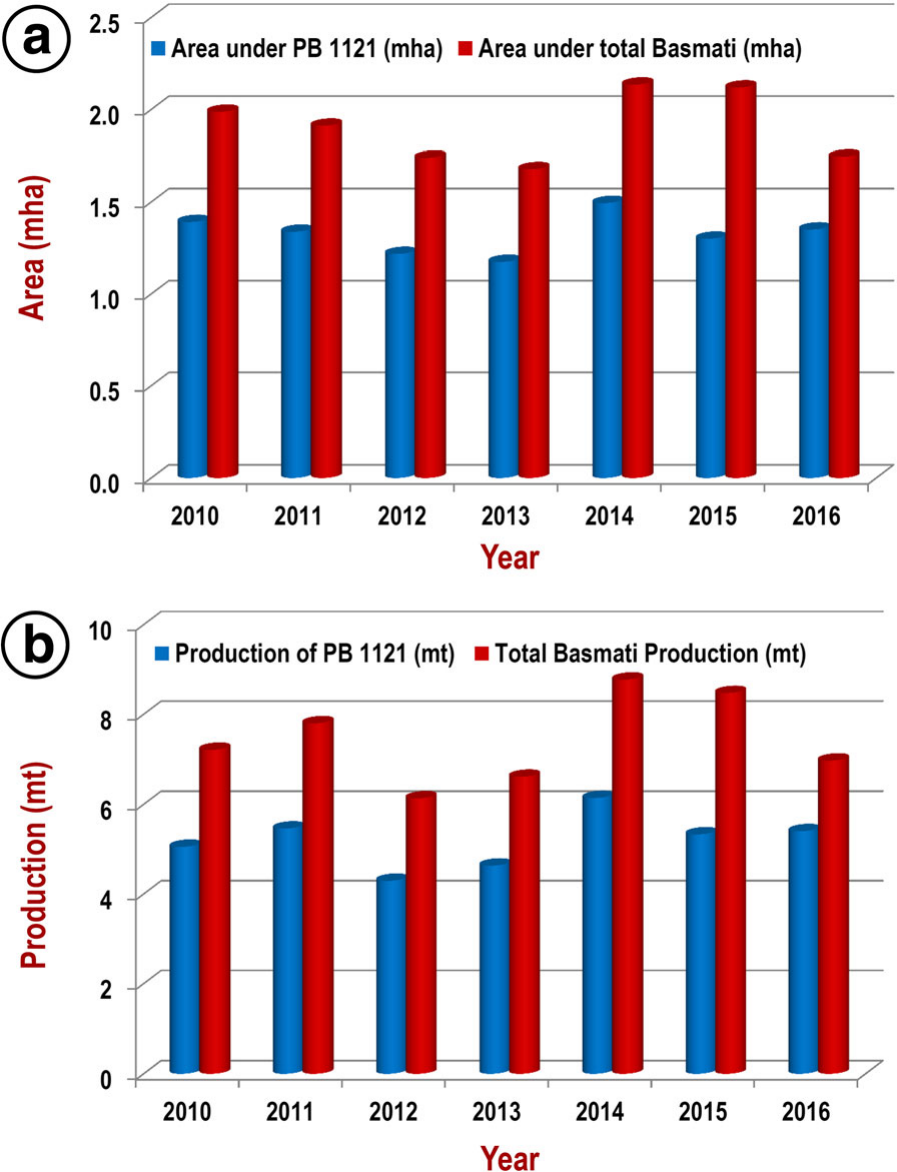
Area (**a**) and yield (**b**) of Pusa Basmati 1121 compared to the totals for all Basmati rice

Owing to its higher yield and unique grain and cooking quality traits, PB 1121 received phenomenal acceptance in the domestic as well as global market encouraging the millers to pay a premium price to the farmers. The area under Basmati rice cultivation increased from 0.78 million hectares (mha) in 2004 to 2.12 mha in 2015, in which the PB1121 is cultivated on about 70% of the total area (Fig. 6a, Additional file 1: Table S1). The total Basmati rice produced during *Kharif* 2016 was 6.16 million tones (mt), of which PB 1121 contributed 4.39 mt (~ 68%) (Fig. 6b, Additional file 1: Table S1). Average net income due to cultivation of PB 1121 is about US$ 1400/ha compared to US$ 650/ha from traditional Basmati rice varieties such as Basmati 370 and Taraori Basmati in 2017 (Unpublished data).

**Fig. 7:**
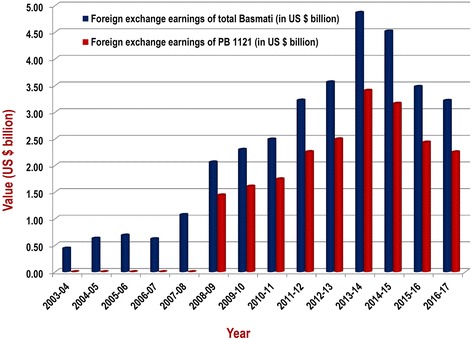
Trends in foreign exchange earnings through export of Basmati rice since the release of Pusa Basmati 1121 (Source: http://agriexchange.apeda.gov.in/indexp/reportlist.aspx)

**Fig. 8:**
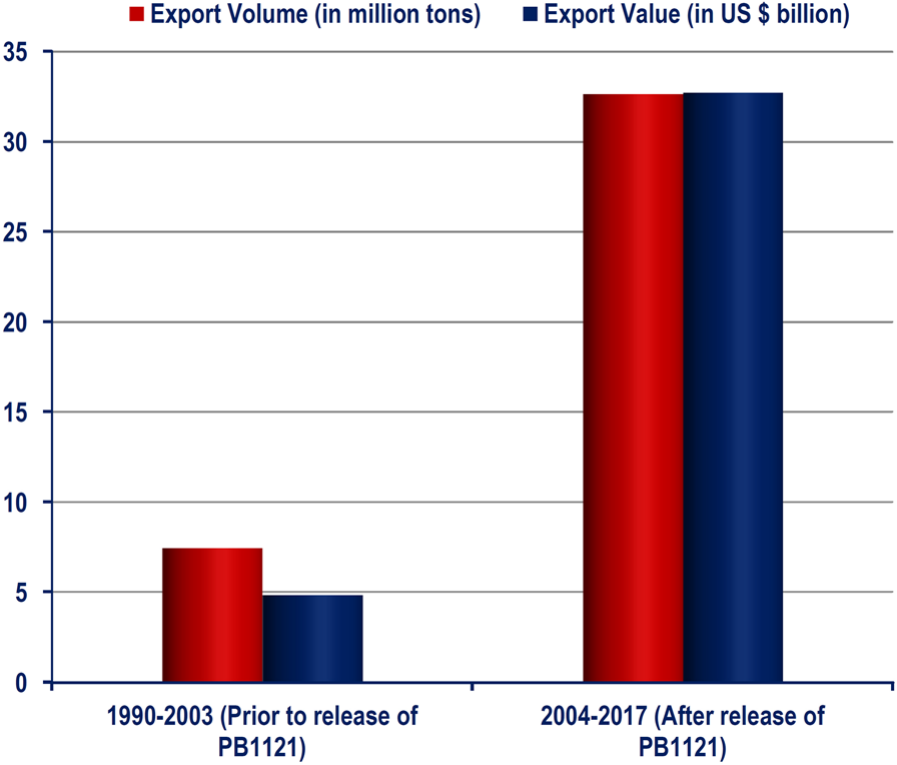
Trend in Basmati export (volume and value) through export of Basmati rice in India, before and after the release of PB 1121

Basmati rice fetches a premium price in the international market. The total foreign exchange earning of Basmati rice prior to release of Pusa Basmati 1121 was US$ 440 million (during 2003–04). Owing to the superior cooking quality traits of Pusa Basmati 1121, the annual foreign exchange earning of Basmati rice has surged during 2013–14 to US$ 4.87 billion, in which the contribution of PB 1121 was US $ 3.41 billion (~ 70%) (Fig. 7). The approximate cumulative earning due to export of PB 1121 and its domestic share from 2008 to 2016 has been estimated to be about US $ 20.80 billion. An analysis of the impact of PB 1121 on Basmati exports shows that while the volume of Basmati rice exports has increased by 4.4 times, the value has increased by 6.8 times after the release of PB 1121 compared to the same period before the release of PB 1121 (Fig. 8).

## PB 1121 in genetic and molecular mapping

**Fig. 9:**
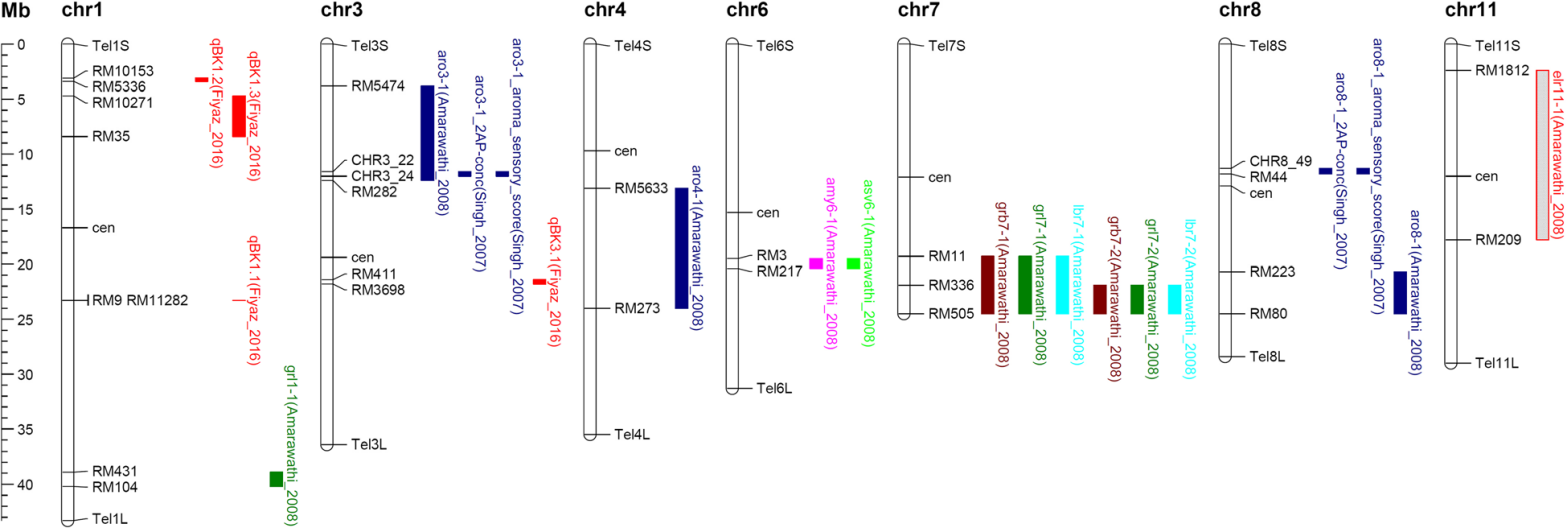
QTLs mapped for various traits in different chromosomes using PB 1121 as one parent in development of a mapping population

PB 1121 provides unique phenotypes for milled rice kernel length and linear cooked kernel elongation. Therefore, it has been used by various researchers in developing populations for the mapping and validation of quantitative trait loci (QTL) governing grain length, cooked kernel elongation, aroma, and other traits, which is summarized in Table 2 and Fig. 9. Amarawathi et al. (2008) mapped QTLs governing grain length on chromosomes 1 (*grl1–1*) and 7 (*grl7–1*) using a recombinant inbred line (RIL) population from the cross PB 1121/Pusa 1342, which were further fine mapped to a region of 108 kb and 2.39 Mb, respectively (Singh et al. 2012). QTL mapping using an F_2_ population from a short grain aromatic rice genotype, Sonasal and PB 1121 identified another major QTL on chromosome 3, which co-localized with *GS3*. Molecular analysis using a functional marker, SF28 for the gene *GS3*, revealed the presence of a C-A mutation in exon 2 in PB 1121 (Singh et al. 2011). Three QTLs governing aroma, *aro3–1*, *aro4–1* and *aro8–1* were identified using the same population, out of which *aro3–1* and *aro8–1* were fine mapped to 390 kb and 430 kb intervals (Singh et al. 2007). Based on whole genome re-sequencing, we have confirmed the presence of an 8 bp deletion in the *Badh2* locus governing aroma in PB 1121. It is highly susceptible to bakanae disease and has been used in mapping a major QTL (*qBK1.2*) governing resistance to this disease of rice (Fiyaz et al. 2016). Among the genes identified for grain quality, *GS3* for grain length and *Badh2* for aroma, and two major QTLs, *qBK1.1*, *qBK1.2* governing resistance to bakanae disease, have been validated and the markers associated with these genes/QTLs are being used in marker assisted improvement of Basmati rice.

**Table 2:** Mapping populations developed using PB 1121 for mapping and validation of QTLs governing various traits in rice

S. No.	Trait(s) mapped	Mapping population (Population used)	QTLs mapped	Marker interval/ linked marker	Percent phenotypic variance (PVE %)	Reference
(a) Mapping of QTLs for grain and cooking quality traits						
1.	Grain length	PB 1121/Pusa 1342 (RILs)	*grl1–1*	RM431-RM104	10.10	Amarawathi et al. 2008
			*grl7–1*	RM11-RM505	7.40	
			*grl7–2*	RM505-RM336	5.70	
2.	Grain breadth	PB 1121/Pusa 1342 (RILs)	*grb7–1*	RM11-RM505	10.10	
			*grb7–2*	RM505-RM336	18.90	
3.	Length/ breadth ratio	PB 1121/Pusa 1342 (RILs)	*lbr7–1*	RM11-RM505	10.00	
			*lbr7–2*	RM505-RM336	21.90	
4.	Elongation ratio	PB 1121/Pusa 1342 (RILs)	*elrl11–1*	RM1812-RM209	6.80	
5.	Amylose content	PB 1121/Pusa 1342 (RILs)	*amy6–1*	RM3-RM217	39.60	
6.	ASV	PB 1121/Pusa 1342 (RILs)	*asv6–1*	RM3-RM217	6.90	
7.	Aroma	PB 1121/Pusa 1342 (RILs)	*aro3–1*	RM5474-RM282	10.30	
			*aro4–1*	RM5633-RM273	6.10	
			*aro8–1*	RM223-RM80	18.90	
8.	Resistance to bakanae disease^a^	PB 1121/Pusa 1342 (RILs)	*qBK1.1*	RM9-RM11282	6.49	Fiyaz et al. 2016
			*qBK1.2*	RM10153-RM5336	24.70	
			*qBK1.3*	RM10271-RM35	4.70	
(b) Fine mapping of QTLs and allele mining						
9.	Aroma	PB 1121/Pusa 1342 (RILs)	*aro3–1*	CHR3_22-CHR3_24	11.00	Singh et al. 2007
			*aro8–1*	CHR8_49-RM44	2.90	
10.	Grain length	PB 1121/Pusa 1342 (RILs)	*qGRL7.1*	CHR7_34-RM505	15.20	Singh et al. 2012
			*qGRL1.1*	CHR1_1-RM431	10.80	
			*qGRB7.1*	CHR7_34-RM505	8.60	
			*qLBR7.1*	CHR7_34-RM505	8.60	
11.	Grain length	Sonasal/PB 1121 (RILs)	Novel InDel in *GS3*	aks-GS3–12	–	Anand et al. 2015
(c) Validation of QTLs						
12.	Grain length	Sonasal/PB 1121 (F_2_)	*GS3*	SF28	32.50	Anand et al. 2013
			*GS3*	SR17	7.90	
			*GS3*	RGS1	12.00	
			*GS3*	RGS2	6.30	
15.	Grain length	Sonasal/PB 1121 (F_2_)	*qgrl7.1*	RM505	5.40	Anand et al. 2013

^a^ PB 1121 is used as a susceptible parent

## Genomic characterization of PB 1121

Whole genome sequencing using next-generation sequencing technologies helps in the discovery of key polymorphisms in crops such as rice, where a high quality reference sequence is available (Krishnan et al. 2012). The whole genome of PB 1121 was sequenced using Illumina Hiseq 2500 platform. A total of 124 million high quality reads with an average read length of 100 bp were obtained, and the average sequencing depth was 50X. We mapped 122.29 million (98.03%) reads to the *Oryza sativa* L. cv. Nipponbare reference genome. A total of 82.2 million reads were uniquely mapped to chromosomes, 0.53 million reads possessed secondary alignments and 1.92 million reads were unmapped.

A total of 3,036,468 variants (2,765,215 SNPs and 292,003 InDels) were identified with average variant rate ranging from 91 bp on chromosome 10 to 163 bp on chromosome 2 (Additional file 1: Table S2). The number of transitions was comparatively higher than that of transversions with *Ts*/*Tv* ratio of 2.50. The annotation of variants revealed 56.24% of variants to be missense and 2.10% of variants to be nonsense, and the remaining 41.66% were silent mutations. A total of 85.54% of the variations were found in non-genic regions while, 14.46% of variations were found in genic regions. PB 1121 possesses extra-long slender grains along with strong aroma and alkali spreading value of 7. Haplotype analysis revealed presence of the long grain specific haplotype with CTGAAG in the second exon of *GS3*, which governs grain length in rice; the 8 bp deletion was found in *Badh2*, which governs aroma; and a haplotype of G_6752756_-T_6752887_T_6752888_ was found in the *ALK* gene which governs alkali spreading value of rice.

## Important varieties and genotypes developed using PB 1121 as a parent

**Fig. 10:**
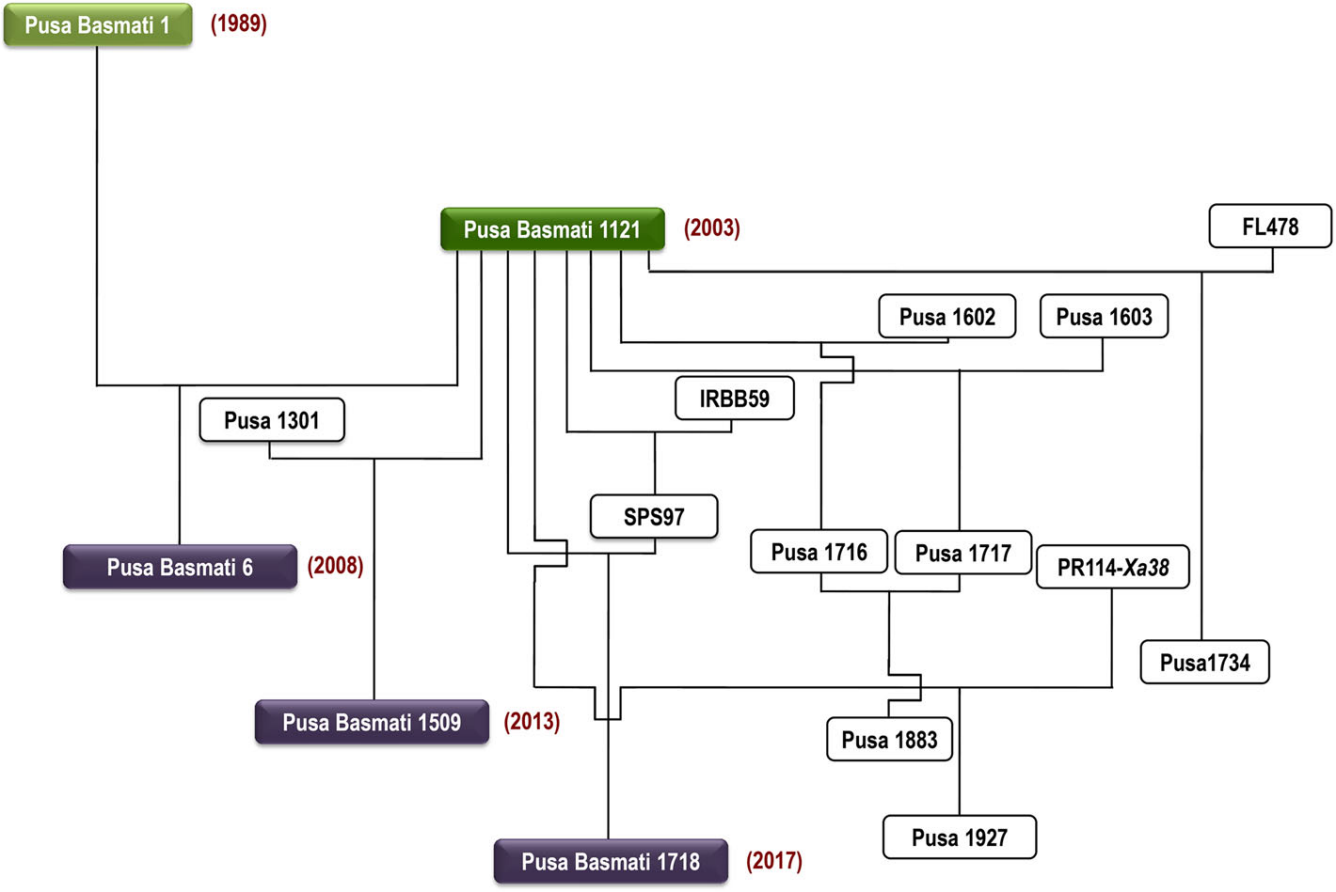
Important Basmati rice varieties and improved Basmati rice genotypes derived from PB 1121

PB 1121 has been used extensively as a parent in conventional breeding to develop better Basmati rice varieties including Pusa Basmati 6 and Pusa Basmati 1509 which have been released for commercial cultivation in the years 2008 and 2013, respectively (Fig. 10).

### Pusa Basmati 6

Also known as Pusa 1401, it has semi-dwarf plant stature with sturdy stem and yields up to 7 t/ha in farmers’ fields. The cooked kernel of Pusa Basmati 6 has uniform shape, as compared to that with a tapering end in PB 1121. It also possesses strong aroma and minimum chalkiness (< 4%).

### Pusa Basmati 1509

A semi-dwarf early maturing Basmati rice variety, which has been released for the national capital region of Delhi, Uttar Pradesh and Punjab (Singh et al. 2014). Pusa Basmati 1509 has a semi-dwarf plant stature (plant height of 95–100 cm), and therefore it is resistant to lodging. It has a 120 days seed to seed maturity, the shortest duration for any Basmati rice variety released so far. The variety produces an average yield of 4.1 t/ha with yield levels as high as 7.0 t/ ha under good management. Quality wise, this variety possesses aromatic extra-long slender grains (8.41 mm) with very occasional grain chalkiness, very good kernel length after cooking (19.1 mm), desirable ASV (7.0) and intermediate amylose content (21.2%).

### Pusa Basmati 1718

Pusa Basmati 1718 (PB 1718) is a marker-assisted selection (MAS) derived near-isogenic line of PB 1121 possessing two genes governing resistance to BB disease, *xa13* and *Xa21* (Singh et al. 2018). It has a seed to seed maturity of 136–138 days and average yield of 4.64 t/ ha. It has been released and notified for the states of Punjab, Haryana and Delhi of the Basmati growing region of the country. It shows resistance to BB with a susceptibility index (SI) of 2 as compared to 7.7 of PB 1121, which is highly susceptible in Basmati growing regions. PB 1718 possesses long slender grains (8.1 mm) with very little grain chalkiness, very good kernel length after cooking (17.0 mm), intermediate amylose content (22.2%) and strong aroma.

Since, PB 1121 occupies a major share in Basmati rice cultivation and export, ICAR-IARI has a major program on improving this variety for resistance to different biotic stresses, namely, BB, blast and abiotic stresses such as seedling stage salt tolerance through precise transfer of gene(s)/QTLs governing these traits with the aid of molecular marker assisted improvement (Table 3).

**Table 3:** Basmati rice varieties developed using PB 1121, along with characteristic features of the trait (genes incorporated) that has been improved

S. No.	Name of the Basmati Rice Variety/ Genotype	Year of Release	Pedigree	Special attribute(s)	Area for which it is Recommended for Cultivation
Released varieties					
	Pusa Basmati 6	2008	Pusa Basmati 1/PB 1121	Milled rice less than 4% chalky grains, cooked rice uniform in shape, strong aroma	Basmati growing areas of Punjab, Haryana, Delhi, Uttarakhand and Uttar Pradesh
	Pusa Basmati 1509	2013	Pusa 1301/PB 1121	Semi-dwarf early maturing Basmati rice variety with a seed to seed maturity of 120 days with grain quality comparable to PB 1121	Basmati growing areas of Uttar Pradesh, Punjab and Delhi (Singh et al. 2014)
	Pusa Basmati 1718	2017	PB 1121/SPS 97// PB 1121*3	A MAS derived bacterial blight resistant near-isogenic line of PB 1121 possessing two genes governing resistance to bacterial blight disease, *xa13* and *Xa21*	Basmati growing areas of Punjab, Haryana and Delhi (Singh et al. 2018)
Improved PB 1121 genotypes					
	Pusa 1883	–	Pusa 1716/Pusa 1717	A MAS derived blast resistant near-isogenic line of PB 1121 possessing two genes governing resistance to blast disease namely, *Pi2* and *Pi54*	Not yet released. (Ellur et al. 2016)
	Pusa 1927	–	PB 1121/PR114-*Xa38*//PB 1121*2	A MAS derived bacterial blight resistant near-isogenic line of PB 1121 possessing BB resistance gene, *Xa38* governing resistance to bacterial blight disease	Not yet released. (Ellur et al. 2016)
	Pusa 1734	–	PB 1121/FL478/PB 1121*3	A MAS derived near-isogenic line of PB 1121 possessing “*Saltol*”, a major QTL governing tolerance to salt stress at seedling stage	Not yet released. (Babu et al. 2017)

## Conclusions

Overall, the Basmati rice research work carried out at ICAR-IARI has resulted in reducing the crop duration from 160 days in traditional Basmati to 140–145 days in PB 1121, combined with doubling the yield from 2.5 t/ha to nearly 5.0 t/ha. This has immensely helped in creating employment in the Basmati growing region, which has resulted in the establishment of more modern rice mills and making available better quality Basmati rice for the domestic and global market on a regular basis. It has greatly improved the economics of Basmati rice cultivation, and also the entire value chain of the Basmati rice industry, especially exporters and consumers thereby ushering in a Basmati rice revolution.

However, Basmati rice cultivation is facing several challenges from diseases such as bacterial blight, bakanae, neck blast, and insect pests like brown plant hopper and stem borer. Therefore, breeding Basmati cultivars with resistance to these diseases, pests and other abiotic stresses while maintaining the superior grain quality by reducing chalkiness, improving HRR and better aroma is the major focus for Basmati rice improvement.

In this endeavour, PB 1718, a bacterial blight resistant near isogenic line of PB 1121, has been developed and released for commercial cultivation in 2017 (Singh et al. 2018). In order to sustain Basmati rice cultivation, it is pertinent to continuously improve the iconic rice variety such as PB 1121, and efforts are already underway to pyramid resistance to bacterial blight, blast and bakanae diseases. The whole genome re-sequencing of PB 1121 has been accomplished which will help in better understanding the genetic and molecular basis of the complex nature of the Basmati grain and cooking quality in PB 1121, and open up new areas of research in Basmati rice, thereby providing an altogether new dimension to Basmati rice breeding. Pyramiding gene(s)/QTL(s) governing resistances to major biotic and tolerance to abiotic stresses, while retaining the premium grain and cooking quality of Basmati rice will go a long way in overcoming the problem of potential risks of the pesticide residue and reducing the cost of cultivation.

## Additional file

Additional file 1: **Table S1** Area and production of total Basmati rice *vis~a~vis* PB 1121. **Table S2** Panel test scores of PB 1121 as compared to Taraori Basmati and Pusa Basmati 1. **Table S3** Reads mapped and variants discovered from whole genome re-sequencing of PB 1121. (DOC 61 kb)

## Abbreviations

ASV: Alkali spreading value
BB: Bacterial blight
GI: Geographical Indication
HBC 19: Haryana Basmati Collection 19
ICAR-IARI: ICAR- Indian Agricultural Research Institute
IET: Initial Evaluation Trial
InDels: Insertion and Deletions
IRRI: International Rice Research Institute
IRRN: International Rice Research Newsletter
L/B: Length/ Breadth
MAS: Marker assisted selection
mha: Million hectares
mt: Million tones
PB 1121: Pusa Basmati 1121
PB 1718: Pusa Basmati 1718
QTL: Quantitative trait loci
RIL: Recombinant inbred line
SNPs: Single nucleotide polymorphisms
