# Supplementary material for: Pusa Basmati 1121 – a rice variety with exceptional kernel elongation and volume expansion after cooking
Source: Rice (N Y). 2018 Apr 9;11:19. doi: 10.1186/s12284-018-0213-6 (PMC5890003; doi:10.1186/s12284-018-0213-6)
Supplement: Supplementary file 1 — Table S1 Area and production of total Basmati rice vis~a~vis PB 1121. Table S2 Panel test scores of PB 1121 as compared to Taraori Basmati and Pusa Basmati 1. Table S3 Reads mapped and variants discovered from whole genome re-sequencing of PB 1121. (DOC 61 kb) [file 12284_2018_213_MOESM1_ESM.doc]

**Additional information:**

**Table S1** Area and production of total Basmati rice *vis~a~vis* PB 1121.

**Table S2** Panel test scores of PB 1121 as compared to Taraori Basmati and Pusa Basmati 1.

**Table 3** Reads mapped and variants discovered from whole genome re-sequencing of PB 1121.

**Table S1** Area and production of total Basmati rice vis~a~vis PB 1121.

| **Year** | **Area under PB 1121 (mha)** | **Total area under Basmati (mha)** | **Production of PB 1121 (mt)** | **Total Basmati production (mt)** |
| --- | --- | --- | --- | --- |
| 2010 | 1.22 | 1.99 | 4.87 | 7.21 |
| 2011 | 1.35 | 1.91 | 5.85 | 7.80 |
| 2012 | 1.36 | 1.73 | 5.76 | 7.11 |
| 2013 | 1.27 | 1.68 | 5.04 | 6.62 |
| 2014 | 1.16 | 2.13 | 4.59 | 8.72 |
| 2015 | 1.34 | 2.12 | 5.09 | 8.06 |
| 2016 | 1.23 | 1.69 | 4.39 | 6.16 |

**Table S2** Panel test scores of PB 1121 as compared to Taraori Basmati and Pusa Basmati 1.

| **Variety** | **Appearance** | **Cohesiveness** | **Tenderness on** | | **Taste** | **Aroma** | **Elongation** | **Overall Acceptability** |
| --- | --- | --- | --- | --- | --- | --- | --- | --- |
|  |  |  | **Touching** | **Chewing** |
| Taraori Basmati | 4.72 | 4.84 | 4.54 | 4.73 | 4.15 | 4.38 | 3.88 | 4.40 |
| Pusa Basmati 1 | 4.35 | 4.07 | 4.41 | 4.45 | 4.00 | 4.01 | 3.64 | 4.06 |
| PB 1121 | 4.89 | 4.57 | 4.75 | 4.64 | 4.14 | 4.39 | 4.43 | 4.45 |
| Range | 4.0-4.9 Creamish white  3.0-3.9  Red streaks | 4.0-4.9  Partially separated  3.0-3.9  Slightly sticky | 4.0-4.9  Moderately soft  3.0-3.9  Moderately hard | 4.0-4.9  Moderately soft  3.0-3.9  Moderately hard | 3.0-3.9  Desirable  2.0-2.9  Tasteless | 4.0 Strong  3.0-3.9 Optimum  2.0-2.9 Mild  1.0-1.9 No scent | 3.0-3.9  Good  2.0-2.9  Moderate | 4.0-4.9  Excellent  3.0-3.9 Good  2.0-2.9 Acceptable |

**Table S3** Reads mapped and variants discovered from whole genome re-sequencing of PB 1121.

| **Chromosome No.** | **No. of reads uniquely mapped** | **Number of Variants** | **Variant rate** |
| --- | --- | --- | --- |
| Chromosome 1 | 9766741 | 347225 | 124 |
| Chromosome 2 | 8301002 | 220169 | 163 |
| Chromosome 3 | 8557509 | 256181 | 142 |
| Chromosome 4 | 7864432 | 269956 | 131 |
| Chromosome 5 | 7075405 | 211572 | 141 |
| Chromosome 6 | 6558590 | 275699 | 113 |
| Chromosome 7 | 6319884 | 256090 | 115 |
| Chromosome 8 | 6169986 | 256904 | 110 |
| Chromosome 9 | 4941784 | 170326 | 135 |
| Chromosome 10 | 5100497 | 252589 | 91 |
| Chromosome 11 | 5617879 | 309923 | 93 |
| Chromosome 12 | 5926940 | 230584 | 119 |
